# Meiofauna increases bacterial denitrification in marine sediments

**DOI:** 10.1038/ncomms6133

**Published:** 2014-10-16

**Authors:** S. Bonaglia, F. J. A Nascimento, M. Bartoli, I. Klawonn, V. Brüchert

**Affiliations:** 1 Department of Geological Sciences, Stockholm University, 106 91 Stockholm, Sweden; 2 Department of Ecology, Environment and Plant Sciences, Stockholm University, 106 91 Stockholm, Sweden; 3 Department of Life Sciences, University of Parma, 43124 Parma, Italy

## Abstract

Denitrification is a critical process that can alleviate the effects of excessive nitrogen availability in aquatic ecosystems subject to eutrophication. An important part of denitrification occurs in benthic systems where bioturbation by meiofauna (invertebrates <1 mm) and its effect on element cycling are still not well understood. Here we study the quantitative impact of meiofauna populations of different abundance and diversity, in the presence and absence of macrofauna, on nitrate reduction, carbon mineralization and methane fluxes. In sediments with abundant and diverse meiofauna, denitrification is double that in sediments with low meiofauna, suggesting that meiofauna bioturbation has a stimulating effect on nitrifying and denitrifying bacteria. However, high meiofauna densities in the presence of bivalves do not stimulate denitrification, while dissimilatory nitrate reduction to ammonium rate and methane efflux are significantly enhanced. We demonstrate that the ecological interactions between meio-, macrofauna and bacteria are important in regulating nitrogen cycling in soft-sediment ecosystems.

## Abstract (web summary)

Excessive nutrient loading is a threat to aquatic ecosystems; however, denitrification may be key in removing large amounts of reactive nitrogen and, therefore, mitigating consequent eutrophication. Here, the authors explore how meiofauna may impact the rate of denitrification in sediments.

## Graphical abstract

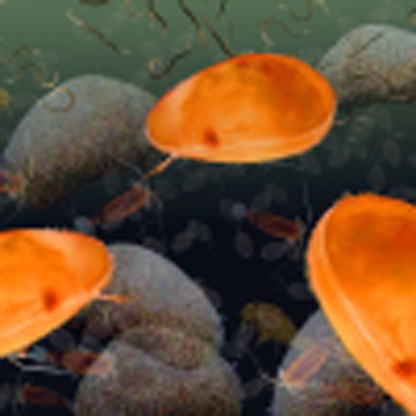

Markedly increased nitrogen (N) loading of many coastal aquatic environments worldwide has had negative global ecological and economical consequences for biodiversity and water quality^1^,^2^. Denitrification is a potentially important ecosystem process in coastal sediments that experience high anthropogenic N loads, because it is estimated to remove globally ~24 Tg of fixed N from the system per year^3^. Although denitrification occurs globally, aquatic environments are far more important N sinks than terrestrial ones^4^. Sediments are preferential places for denitrification, because they are often characterized by low oxygen (O_2_) concentrations, sharp oxic/anoxic interfaces and high rates of nitrate (NO_3_^−^) and organic matter supply^4^.

Besides denitrification, two other pathways of nitrate reduction have been shown to play a role in marine sediments: anammox—the anaerobic oxidation of ammonium (NH_4_^+^) to dinitrogen (N_2_) by reduction of nitrite (NO_2_^−^), and the dissimilatory nitrate reduction to ammonium (DNRA)^5^. Generally, anammox accounts for less than 20–30% of the total benthic N_2_ production in shallow coastal environments^6^, while DNRA can be more important than denitrification in coastal and estuarine areas^7^,^8^. DNRA retains fixed nitrogen in the environment and further enhances eutrophication when denitrification is outcompeted^5^,^9^. Given the magnitude of the ecological and economic problems caused by eutrophication, understanding the mechanisms that control nitrogen removal from aquatic ecosystems remains a central point in ecology, with clear environmental policy and management implications^2^,^10^. However, the complexity of benthic N cycling, in particular of the multiple nitrate reduction pathways, is hard to tackle in field studies, and experimental investigations of feedbacks between trophic levels and their effects on ecosystem processes are needed^11^.

Benthic macrofauna (invertebrates >1 mm) is widely recognized to play an important role in the regulation of carbon (C) mineralization, nutrient regeneration and coupled nitrification/denitrification^12^,^13^. Macrofaunal activity is generally known to enhance denitrification due to particle reworking and burrowing, ventilation and bioirrigation (refs 14, 15 and references therein) whereas, in some cases, it can negatively impact denitrification and enhance N recycling by means of DNRA stimulation^16^. Most studies dealing with the effects of fauna on benthic biogeochemistry have considered large animals because they are easy to manipulate in the laboratory and are expected to physically alter microbial pathways and process rates limited by diffusive supply or other constraints (for example, sediment aging, burial to strictly anoxic zones or exhaustion of energy-yielding electron acceptors)^16^,^17^,^18^.

While there is a vast body of literature dealing with macrofauna and its effect on sediment biogeochemistry^12^,^14^, only a few papers deal with the role of other potentially important benthic organisms such as meiofauna (benthic animals between 0.04 and 1 mm) on benthic ecosystem services^19^,^20^. Meiofauna is the most abundant and diverse metazoan group in aquatic sediments^21^ and corresponds to ~60% of total metazoan abundance on Earth^22^. Moreover, in benthic environments with low input of organic matter where endobenthic macrofauna abundance is low, meiofauna is often not only the most abundant but also the faunal group with the highest biomass^23^,^24^,^25^. Interactions between meiofauna and sediment prokaryotes have recently been shown to have an important effect on benthic ecosystem processes such as organic matter mineralization^19^ or degradation of organic pollutants^20^ and only a few authors have acknowledged the importance of meiofauna for solute transport and oxygen and nutrient cycling^24^,^26^,^27^. Recently, it has also been shown that certain meiofaunal groups (foraminifera) are even capable of complete denitrification^28^,^29^, but the general role of meiofauna for benthic nitrogen cycling remains poorly understood.

Here, we test whether microbioturbation by meiobenthos and meiofauna–macrofauna interactions have a significant effect on (1) pathways of relevant electron acceptors (oxygen and nitrate) in surface sediments and on (2) fluxes of end products of anaerobic metabolism, such as methane. We show that meiofauna positively affects nitrification and denitrification, thus enhancing sedimentary nitrogen loss. On the other hand, macrobenthic bivalves increase N recycling by stimulating DNRA and the efflux of methane. Biological interactions between meio-, macrofauna and bacteria are therefore important factors that regulate essential benthic biogeochemical processes such as nitrogen loss and methane release.

## Results

### Infauna abundances and community structure

This experiment had four main treatments with changing infaunal composition. It included two treatments with high meiofauna abundance and diversity: HM, high meiofauna and no macrofauna, and HMM, high meiofauna+the macrofaunal bivalve *Macoma balthica*; plus two treatments with low meiofauna abundance and diversity: LM, low meiofauna and no macrofauna, LMM, low meiofauna+*M. balthica.* In addition, we also included a treatment of undisturbed and unmanipulated sediment cores (CTRL) with the natural infaunal community composition and structure.

The extraction methodology established a large difference in meiofauna abundances between the treatments with low meiofauna (LM and LMM) and the two treatments with high meiofauna (HM and HMM). The meiofauna organisms remaining in the LM and LMM treatments were predominantly small nematodes (on average 86 and 74 ind. 10^−3^ m^−2^, respectively) and ostracods (15 and 16 ind. 10^−3^ m^−2^, respectively; Table 1). The HM and HMM treatments contained a more diverse meiofauna community with high abundances of nematodes (718 and 680 ind. 10^−3^ m^−2^, respectively) and ostracods (44 and 38 ind. 10^−3^ m^−2^, respectively) together with copepods, kinorynchs and low abundances of oligochaetes, which resulted in abundances on average seven times higher than in the LM and LMM treatments (Table 1). When compared with the control treatment, meiofauna abundance was significantly higher in the HM and HMM and lower in the LM and LMM treatment (analysis of variance (ANOVA), *P*<0.0001), and this was mainly due to differences in nematode abundances (Table 1). However, the meiofauna abundances in the HM, HMM and unmanipulated control treatment were within the same order of magnitude reported for the field area where the sediment was collected^30^.

All macrofauna specimens that we added to the LMM and HMM treatments were recovered alive at the end of the experiment, that is, two individuals of *M. balthica* in each sediment core, corresponding to ~2,000 ind. m^−2^ and a biomass of 1.92 g C m^−2^. The unmanipulated control treatment was found to contain individuals of both *Marenzelleria* spp. (Polychaeta) and *M. balthica*, with *Marenzelleria* reaching higher densities than the bivalve (~1,400 and 400 ind. m^−2^, respectively) as well as higher biomasses (0.97 versus 0.38 g C m^−2^, respectively).

### Concentration gradients and gas fluxes

**Figure 1:**
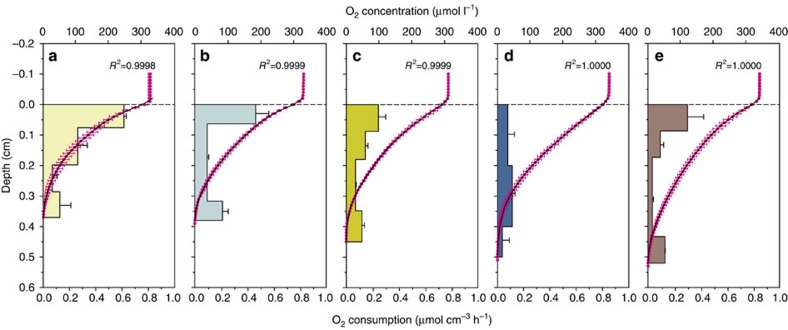
Measured oxygen concentration profiles and calculated oxygen consumption profiles. Average oxygen (O_2_) concentration profiles measured in the different treatments are reported in magenta. Black curves are the best fitting profiles calculated using the numerical interpretation method by Berg *et al.*^65^ Horizontal bars represent the depth average oxygen consumption rates and result from the modelling procedure. (**a**) low meiofauna; (**b**) high meiofauna; (**c**) low meiofauna+macrofauna; (**d**) high meiofauna+macrofauna; (**e**) unmanipulated sediment cores. Error bars represent s.d. (*n*=6 per treatment).

The average O_2_ penetration depth and concentration profiles of O_2_ are reported in Table 2 and Fig. 1, respectively. The average penetration depths from the different treatments were organized as LM<HM<LMM<HMM<CTRL with significant differences among treatments (ANOVA, *P*<0.001, Table 3). High meiofauna abundance did not significantly enhance O_2_ penetration compared with low meiofauna abundance, whereas the presence of *M. balthica* increased O_2_ penetration depth (LMM, HMM, CTRL; Table 3 and Fig. 1).

The theoretical molecular diffusive O_2_ flux (*J*_diff_), calculated without taking into consideration biodiffusivity, decreased in the order HMM<LMM<HM<LM (Table 2). The CTRL treatment was included to determine how the processes measured in our study occur in unmanipulated intact sediment. As the remaining treatments intended to vary meio- and macrofauna abundances, they are not directly comparable to the CTRL treatment. Molecular diffusivity (*D*_S_) was very similar among treatments (range 9.9 to 10.7 × 10^−6^ cm^2^ s^−1^) and reflected the narrow range of porosities in the different treatments (Table 2). The total oxygen flux (*J*_tot_), measured by whole-core incubation and representing the sum of the O_2_ consumption due to infauna activity and *J*_diff_, was significantly different among treatments (ANOVA, *P*=0.003, Table 3) and decreased in the order LM<LMM<HM<HMM (Table 2). Biodiffusivity (*D*_B_) was dependent on the biomass of the animals in each treatment and followed the same trend as the total oxygen flux, with the lowest *D*_B_ recorded in LM (2.4 × 10^−6^ cm^2^ s^−1^) and the highest in HMM (27.2 × 10^−6^ cm^2^ s^−1^) (Table 2).

**Figure 2:**
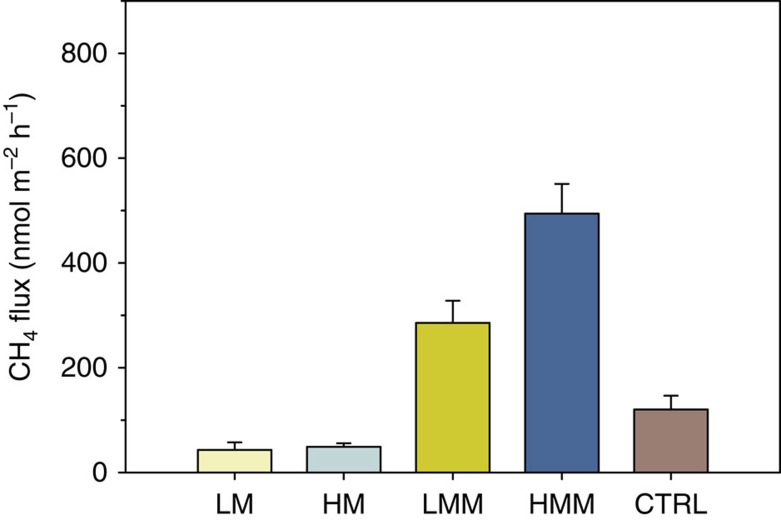
Fluxes of methane in the different treatments. Vertical bars represent average fluxes of methane (CH_4_) determined by intact-core incubations. CTRL, unmanipulated sediment cores; HM, high meiofauna; HMM, high meiofauna+macrofauna; LM, low meiofauna; LMM, low meiofauna+macrofauna. Error bars represent s.e.m. (*n*=8 per treatment).

Methane (CH_4_) efflux to the water column was significantly different among the treatments (Kruskal–Wallis, *P*<0.001, Table 3), with significantly higher emissions associated with sediments inhabited by bivalves when compared with those with only meiofauna, irrespective of whether they had high or low abundances and diversity (Table 3 and Fig. 2). The control treatment showed an intermediate situation, with CH_4_ fluxes ranging between HM and LMM. HMM showed a CH_4_ efflux 10 times higher than HM, whereas in LMM the CH_4_ efflux was seven times higher than in LM, suggesting that bivalve activity coupled to high meiofauna abundance and diversity either stimulated methanogenesis or methane transport. The higher effluxes of CH_4_ in HMM and LMM when compared with CTRL, which was dominated by *Marenzelleria* spp., suggest that *M. balthica* stimulated CH_4_ emission over *Marenzelleria* spp.

### Denitrification and DNRA rates

**Figure 3:**
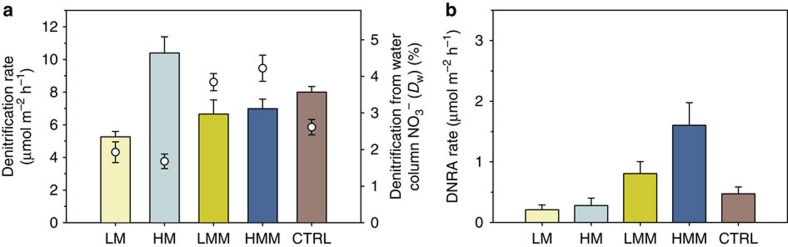
Rates of denitrification and DNRA in the different treatments. (**a**) Average rates of denitrification (vertical bars) and contribution of denitrification from water column nitrate (*D*_w_) to total denitrification (open circles) and (**b**) average rates of DNRA in the different treatments determined from intact-cores amended with ^15^N-nitrate. CTRL, unmanipulated sediment cores; HM, high meiofauna; HMM, high meiofauna+macrofauna; LM, low meiofauna; LMM, low meiofauna+macrofauna. Error bars represent s.e.m. (*n*=7 per treatment).

The isotope pairing technique (IPT) makes it possible to divide total N_2_ production (*D*_tot_) into the contribution of denitrification coupled to nitrification (*D*_n_) and denitrification based on water column NO_3_^−^ (*D*_w_) (ref. 31). Denitrification rates were more than 95% due to *D*_n_ (Fig. 3a). HM had significantly higher denitrification rates than the other treatments (ANOVA, *P*<0.001, Table 3). In the HM treatment, denitrification was twofold higher than in LM, 60% higher than LMM and 50% higher than HMM. *D*_w_ was significantly higher in the macrofauna treatments (LMM and HMM) compared with the treatments with only meiofauna (LM and HM; Table 3). Labelled N_2_ concentrations in Exetainers incubated with anoxic water amended with ^15^NO_3_^−^ were not significantly different between controls (filtered water) and vials with increasing densities of nematodes (ANOVA, *P*>0.05) indicating insignificant denitrification by the nematodes themselves. Tests with anoxic sediment slurries incubated with ^15^NH_4_^+^+^14^NO_3_^−^ did not produce labelled N_2_, which suggest that anammox did not significantly contribute to N_2_ production in sediments taken from our sampling area.

For all treatments, rates of nitrate reduction to ammonium (DNRA) were lower than denitrification rates (Fig. 3b). However, the proportion between DNRA and *D*_tot_ varied among treatments and was highest in HMM where DNRA accounted for ~19% of total NO_3_^−^ reduction, followed by LMM (~11%) and the other treatments (<5%). DNRA rates followed the same trend as the CH_4_ fluxes and were significantly higher in sediments inhabited by bivalves (LMM and HMM) compared with those with only meiofauna (LM and HM), irrespective of whether they had a high or low meiofauna abundance (Kruskal–Wallis, *P*=0.004, Table 3). Once again, in the control treatment, DNRA was intermediate between the rates in HM and LMM.

## Discussion

**Figure 4:**
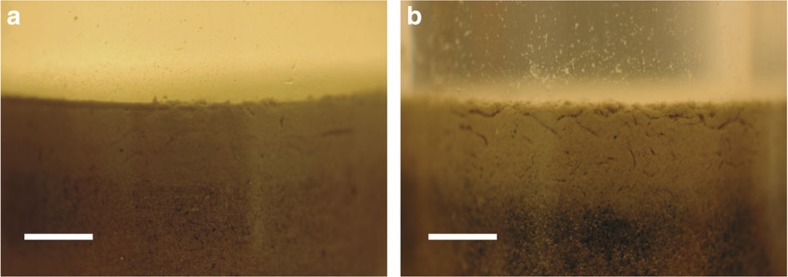
Bioturbation by meiofauna. Digital camera pictures showing the different microbioturbation intensity in the upper sediment layer of microcosms from the low meiofauna treatment (**a**) and the high meiofauna treatment (**b**). Length of scale bars is 500 μm.

Our results show that high meiofauna bioturbation (Fig. 4) enhances the sedimentary production of dinitrogen gas. This enhanced dinitrogen production is not due to direct respiration of nitrate by the meiofauna, which has been shown for some species of foraminifera, a common unicellular meiofaunal group^28^,^29^. In line with previous studies that found foraminifera to be generally present in low abundances in the Baltic Sea^32^, our experimental sediments did not contain significant numbers of individuals of this meiofaunal group. In addition, our incubations with nematodes alone did not result in N_2_ production suggesting that the nematodes (or their symbionts) collected from the anoxic sediment did not have the capacity to denitrify. This observation contrasts with results by Hentschel *et al.*^33^ and indicates that the capacity for eukaryotes for denitrification is species-specific.

These findings indicate that the twofold increase in denitrification rates seen in the HM treatment is due to stimulation of denitrifying bacterial activity rather than direct eukaryotic denitrification. Although certain protists and foraminifera are capable of nitrate respiration^29^,^34^, prokaryotes and especially bacteria are the most representative and widespread denitrifying microorganisms^35^. Aquatic sediments can host both heterotrophic and autotrophic denitrifiers^36^, which are dependent on the availability of nitrate and of a suitable electron donor (for example, organic matter, sulfide and so on). In estuarine and coastal marine sediments, nitrification is often the main source of nitrate for denitrifiers^37^. In the HM treatment *D*_n_, and not *D*_w_, was stimulated compared with the LM treatment, suggesting that nitrification was stimulated by meiofauna activity. It has been previously proposed that meiofauna could promote sedimentary aerobic processes such as nitrification by increasing solute transport and reactions in the oxic zone of the sediments^27^. This is also in line with findings by Prast *et al.*^38^, who found higher abundances of nitrifying bacteria and higher nitrification potential in sediments with ciliates grazing on bacteria.

Studies that investigated meiofauna–bacteria interactions in marine sediments often showed apparently contradictory results: Some authors indicated that meiofauna grazing stimulates the bacterial community^19^,^39^, while others reported the opposite trend, that is, higher meiofaunal densities decrease bacterial activity^20^,^40^. Our data suggest that nematodes, which are the predominant metazoans in marine sediments^41^, stimulate nitrifiers and denitrifiers. Nematodes were by far the most abundant group (~92% of total abundance) in our HM and HMM treatments and have been reported to secrete nitrogen in excess both as inorganic nitrogen (ammonium and nitrate)^42^ and as dissolved organic nitrogen, such as amino acids^43^. In fact, nematodes generally have a higher C:N ratio than bacteria^43^ and grazers on bacteria tend to excrete large quantities of N in their mucus^42^,^43^. We suggest that this process was particularly important in the direct stimulation of nitrification–denitrification as increased availability of ammonium would have stimulated nitrifiers, and increased availability of nitrate and labile organic compounds would have specifically stimulated heterotrophic denitrification. Moreover, in sediments with more active irrigation by meiobenthos transport of solutes like oxygen, ammonium and nitrate is generally increased in animal burrows^26^,^27^ resulting in a microhabitat where the essential substrates for nitrifying and denitrifying bacteria are more available^12^,^15^.

Interestingly, high meiofauna bioturbation did not deepen oxygen penetration significantly. This indicates that meiofauna was mainly active in the uppermost millimeters, as supported by visual inspection (Fig. 4). The oxygen consumption rate calculated from the concentration profiles in the uppermost sediment layer (0–2 mm depth) was consistently lower in HMM and HM than in LMM and LM (Fig. 1). Even when considering biodiffusivity in our numerical interpretations, the O_2_ consumption in this top layer was greater in LMM and LM than in HMM and HM. This may suggest that aerobic microorganisms were more active and abundant in close proximity to the sediment–water interface in low meiofauna than in high meiofauna conditions. Lower predation pressure, when meiofauna density was low, could have enhanced growth of aerobic bacteria and protozoans^44^,^45^. It is also possible that less competition for oxygen in the low meiofauna treatments could have favored aerobic microorganisms.

Somewhat surprisingly, denitrification was lower when *M. balthica* and meiofauna were present in high abundances (that is, HMM versus HM). Macrofauna has been reported to decrease both meiofauna activity and abundance in sediments due to disturbance, predation or competition for food^40^,^46^,^47^. *M. balthica* has recently been shown to reduce meiofauna activity probably due to interference competition for freshly deposited organic matter^48^, while no obvious effect on bacterial abundances has been reported^49^. Thus, it appears that *M. balthica* counteracted the stimulating effect for the nitrifying and denitrifying microbial communities by meiofauna as suggested above. In addition, in sediments with bivalves, nitrification could have been partly inhibited by the presence of sulfides^50^, which could have been mobilized at greater depth by macrofaunal bioturbation^16^. This is supported by the fact that in LMM and HMM we measured the highest rates of DNRA, a process that is tightly coupled to the oxidation of sulfides in sediments^51^. It is likely that all these factors contributed to the decrease in coupled nitrification–denitrification we observed in HMM compared with HM. The treatments with macrofauna had higher *D*_w_ compared with the treatments where *M. balthica* was absent, which agrees with previous studies^17^,^18^.

Macrofaunal bioturbation has previously been shown to restrain the uniform mixing of endogenous ^14^NO_3_^−^ and added ^15^NO_3_^−^ in the denitrification zone because of increased heterogeneity of the sediment due to large polychaete burrow structures^52^. This is a fundamental limitation of the IPT, which may lead to the underestimation of total denitrification activity^31^. Nonetheless, a recent experiment using soft Baltic Sea sediments bioturbated by *Marenzelleria* spp. proved that at high polychaete biomass, the two nitrate isotopes were homogeneously mixed^16^, that is, the significant positive correlation between polychaete biomass and degree of denitrification underestimation^52^ was not observed. In our study, meiofauna likely exerts an opposite effect compared with large polychaetes: the former creates a more homogeneous sediment texture because it freely moves in the interstices^53^ and enhances solute and particle transport within the oxic zone^24^,^26^,^27^. Moreover, since the siphons of *M. balthica* do not secrete mucus and are mostly active in the nitrification zone^54^, its bioturbation may have even helped nitrate mixing.

Anammox is a factor that may lead to the overestimation of total N_2_ production in marine sediments incubated using the IPT^55^. Our tests with anoxic sediment slurries collected from the same geographical area as our experimental sediments and amended with ^15^NH_4_^+^ and ^14^NO_3_^−^ did not result in any significant production of labelled N_2_, suggesting that anammox bacteria were either not active or not present in this shallow coastal sediment. So far, no studies have examined the effect of macro- and microbioturbation on the anammox process. We therefore recommend that future experiments should investigate the effect of faunal bioturbation on anammox in sediments where anammox contributes significantly to N_2_ production rates^6^.

Methane effluxes and DNRA rates showed the same trend among different treatments, with a significantly higher flux/rate in sediments inhabited by bivalves. It is not clear if the increase in methane and ammonium release was due to the presence of symbionts living in the gut of *M. balthica* or to the bioturbation activity by the bivalve. Macrofauna can have anoxic niches inside its gut, which allow the colonization and metabolic activity of anaerobic bacteria^56^. In particular, bivalves can host symbionts in every region of their gut, where fermenting bacteria have been documented^57^. The fact that the bivalves were not found deeper than 1.5–2 cm in our sediments further indicates that there were anoxic microenvironments inside *M. balthica* colonized by nitrate reducers and methanogens, as it has been suggested for other metazoans^58^,^59^. Further investigations are required to examine if bivalves are not only able to consume methane thanks to their symbionts^60^, but also capable of methane production.

The enhancement of nitrification and denitrification by meiofauna suggests that this faunal group can mediate nitrogen cycling in sediments with little or no macrofauna. Indeed, Danovaro *et al.*^41^ has found deep-sea ecosystem functioning and efficiency to be linked to high meiofauna diversity. Meiofauna dominates over macrofauna in terms of biomass, abundance and diversity in low-energy benthic systems^41^, and in systems affected by oxygen depletion^25^. The results presented here show that denitrification is even higher when macrofauna abundance is reduced.

Denitrification by foraminifera can make important contributions to total N_2_ production especially in continental shelves^28^ and deep see sediments^61^. Generally, rates of foraminiferal denitrification may vary between 2.1 and 7.2 μmol N m^−2^ h^−1^ (refs 28, 61). The increase in denitrification rate by meiofauna bioturbation measured in our experiments (5.1 μmol N m^−2^ h^−1^) is even higher than the average denitrification rate by foraminifera suggesting that stimulation of bacterial denitrification by meiofauna activity may be as important as the direct meiofaunal denitrification itself. This is important in light of the strong anthropogenic pressure on benthic ecosystems and their vulnerability to biodiversity loss^62^. Meiofauna has shorter generation times and faster turnover rates than macrofauna^21^, and recovers generally faster from perturbation events related to eutrophication like anoxia or hypoxia^63^. Our findings suggest that meiofauna community recovery could stimulate benthic microbial processes by enhancing biodiffusivity, even if the macrofauna population fails to recover or does so later in time.

Our results demonstrate that meiofauna activity increases the removal of fixed nitrogen from aquatic ecosystems by stimulating nitrification and denitrification in the oxic–anoxic transition zone of the marine sediment. Bivalves stimulate microbial processes as DNRA and net methane release, and this stimulation is also affected by meiofauna bioturbation. By enhancing DNRA, bivalves can to a certain extent counteract the beneficial effects of meiofauna on total N loss. Effects of macrofauna–meiofauna–bacteria interactions on nitrogen transformation processes have been largely unexplored. This study provides important new information on how benthic meiofauna can mitigate environmental problems caused by excessive nitrogen loads in aquatic ecosystems (that is, eutrophication). Further understanding of the mechanisms regulating benthic ecosystem processes such as denitrification requires studies that take into account how ecological interactions between macro-, meio- and microbiological communities of the benthos impact such processes. In particular, it is important to consider other potentially important faunal groups, such as protozoans, in regulating bacteria-mediated processes with important ecosystem function.

## Methods

### Sediment sampling

Sediment cores were collected in July 2012 with a multicorer in Hållsviken (Stockholm Archipelago, Baltic Sea: 58°50′N, 17°32′E) at 28 m depth. Sampling with a multicorer minimizes the ‘bow-wave’ effect on the sediment surface, which can reduce the abundance of epibenthic fauna in the water overlying the sediment. A modified Niskin sampler allowed the collection of bottom water, and a digital multimeter was used to measure temperature (9.4 °C), salinity (6.5) and dissolved oxygen (307 μmol l^−1^). Multicorer sediment liners (*n*=16, i.d. 9 cm, height 60 cm) were subsampled onboard with smaller liners (*n*=45, i.d. 3.6 cm, height 25 cm) to have 12 cm height of sediment and 10 cm of overlying water. The cores were capped with rubber stoppers and transported within 30 min to the Askö Laboratory, Stockholm University Marine Research Center, where they were stored in a cold room and constantly stirred with magnetic bars at *in situ* temperature.

At the same station, additional surface sediment was collected by means of an epibenthic sledge to isolate specimens of *Macoma balthica*, which thereafter were maintained at *in situ* temperature (9.5 °C) in well-aerated aquaria.

### Meiofauna extractions and experimental setup

Meiofauna extractions were carried out the day after sampling using the procedure described in Näslund *et al.*^20^ Briefly, the upper 4 cm of each core were sliced and sieved through a 1,000 and 40-μm sieve. The animals retained in the 1,000-μm sieve were removed as macrofauna, while meiofauna and sediment in the 40-μm sieve were submersed in a MgCl_2_ solution (740 mmol l^−1^) for 5 min to anaesthetize the animals^53^. Meiofauna was separated from the sediment by density extraction using a Levasil colloidal solution (H.C. Starck) with a density of 1.3 kg m^−3^. The extractions were made by shaking an Erlenmeyer flask with sediment and Levasil and let it stand for 5 min while the sediment settled and the animals floated up (settling time). The top part of the solution containing the animals was decanted and washed with seawater (salinity 6.5). This extraction procedure was repeated twice (a second extraction with 5 min of settling time, followed by a third and final extraction with 30 min of settling time). After the last extraction, the sediment retained in the 40-μm sieve was washed thoroughly with seawater to remove the Levasil and reintroduced to the sliced core. The meiofauna individuals extracted from two cores were pooled and added to one of the two experimental units, creating a high meiofauna abundance/diversity. The second experimental unit was left with only extracted sediment and low meiofauna abundance/diversity, that is, the meiofauna that could not be removed by the extraction. After this procedure, we added water with sediment particles that passed through the 40-μm sieve to each experimental unit to reconstitute the finer sediment particle fraction of the original sediments.

A total of 45 microcosms were setup, with five different treatments (*n*=9 per treatment): (1) high meiofauna (HM), microcosms with a high abundance and diversity of extracted meiofauna; (2) high meiofauna abundance and diversity with macrofauna (HMM), identical to HM treatment but with two individuals of *M*. *balthica*; (3) low meiofauna (LM) microcosms with the extracted sediment particles only and no meiofauna addition; (4) low meiofauna with macrofauna (LMM), identical to the LM treatment but with two individuals of *M. balthica* and (5) a control (CTRL) consisting of unmanipulated intact sediment cores. The CTRL treatment was not directly comparable to the other manipulated treatments (HM, HMM, LM and LMM). Its main function was to provide an experimental control, and enable a comparison to field-like conditions.

The abundance of *M. balthica* used in the experimental units of the HMM and LMM treatments corresponds to 2,000 ind. m^−2^, which is similar to densities commonly reported for the sampling area^30^. To keep the fauna alive during the acclimation and incubation time and to re-establish an organic layer on the sediment surface, each experimental unit received a concentrated solution of the green microalgae *Pseudokirchneriella subcapitata* equivalent to ~1.2 g C m^−2^, corresponding to 1–4.5 days of peak sedimentation of phytoplankton material during a spring bloom in the sampling area^30^,^64^.

The sediment cores were subsequently preincubated in a water tank (~150 l) filled with sand-filtered water pumped from 20 m depth. This water was additionally filtered through a 40-μm sieve to remove any possible meiofauna individuals present in the water. The preincubation was carried out in the dark and at constant temperature (9.5 °C) for 10 days. Air stones and water pumps were placed along the walls of the water tank so that the water phase of each microcosm was saturated with oxygen and well mixed. Each microcosm was equipped with a teflon-coated magnet driven by an external rotating magnet (60 r.p.m.) to stir the overlying water in each sediment core. All experiments were carried out in compliance with local ethical regulations.

### Oxygen profiles and benthic diffusive oxygen flux

Oxygen concentration profiles were measured using Clark-type microelectrodes (OX-50, Unisense) in two random sediment cores from each treatment and three to five microprofiles were carried out in each core. The sediment core was transferred to a 10-l aquarium filled with the same water as the incubation tank, so that all the measurements were carried out at the same conditions of temperature, salinity and oxygen saturation. The microelectrode tip (50 μm) was inserted directly inside each sediment core, while an air stone was bubbling air to ensure sufficient water mixing during the measurements.

The recorded concentration profiles were analysed by the numerical interpretation described in Berg *et al.*^65^ This numerical procedure provides the best fit to a measured concentration profile assuming steady state conditions and returns the O_2_ flux between the sediment–water interface as a function of depth. Both molecular diffusivity (*D*_S_) and biodiffusivity (that is, movement of solutes due to bioturbation; *D*_B_) can be included as solute transport mechanisms in the model, and the resulting total O_2_ flux (*J*_tot_) is equal to:


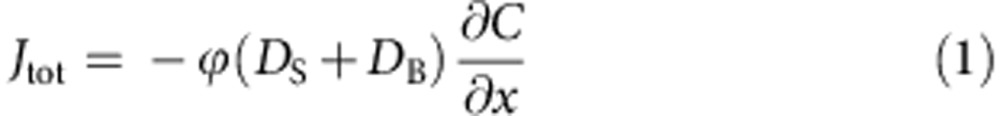


where *φ* is sediment porosity (see below for calculations), *C* is the porewater O_2_ concentration and *x* is depth. In our simulations, we initially set *D*_B_ to zero^26^, so that the molecular diffusive O_2_ flux (*J*_diff_) could be defined as:


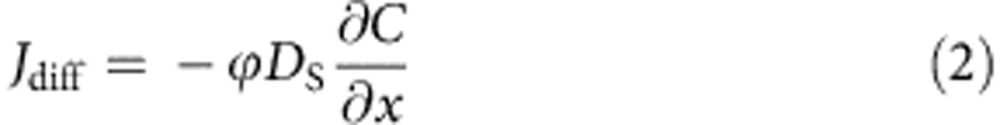


and the *D*_B_ values for the different treatments could be estimated from the relation:


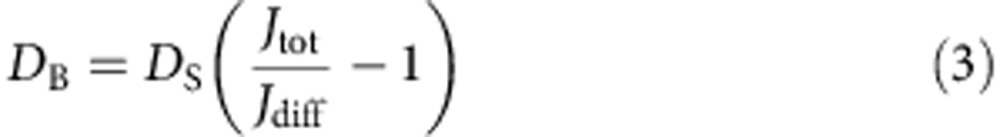


Molecular diffusivity in the sediment (*D*_S_) was calculated according to Iversen and Jørgensen^66^:


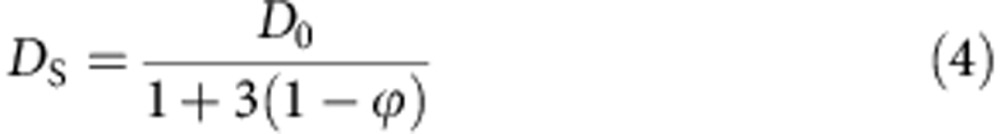


where *D*_0_ is the diffusion coefficient in free water, which was calculated according to Schulz^67^. *J*_tot_ (equation (1)) was measured by means of sediment core incubation (see below for details). In the numerical procedure used to calculate *J*_diff_ (equation 2) the O_2_ concentration at the bottom and the flux at the bottom of the profile were selected as boundary conditions.

### Sediment core incubations

A total of 40 sediment cores (*n*=8 per treatment) with about 100 ml water on top of the sediment were capped with rubber stoppers without headspace gas and stirred with small magnetic stirrers. Incubations were limited to 6 h to ensure that the O_2_ concentration did not decrease below 20% of the initial value. Water samples for CH_4_ were taken at the beginning and the end of the incubation, transferred to 12 ml Exetainer vials (Labco Scientific) and poisoned with 100 μl ZnCl_2_ (7 mol l^−1^). O_2_ concentrations were measured before and after the incubation using a precalibrated minielectrode (OX-500, Unisense).

After the flux incubation, a second incubation was carried out to determine denitrification and DNRA rates according to the IPT^31^. Each sediment core was treated with the same concentration of ^15^NO_3_^−^ to avoid pseudoreplication. Briefly, the water on top of each sediment core was amended with a 20 mmol l^−1^ Na^15^NO_3_ solution (99.3 atom %) to a final concentration of 50 μmol l^−1 15^NO_3_^−^. Samples for NO_3_^−^ analysis were taken before and after ^15^NO_3_^−^ addition to determine the labelling percentage of the NO_3_^−^ pool. The water samples were filtered through 0.45 μm disposable filters and frozen for later analysis. The cores were left uncapped and preincubated for 1.5 h to establish a linear production of ^29^N_2_ and ^30^N_2_ in the sediment. One core per treatment was sacrificed thereafter to measure the background concentration of ^29^N_2_ and ^30^N_2_. The other cores (*n*=35) were capped without headspace and incubated for 6 h while stirring. The incubation was terminated by gently mixing water and sediment in each core to slurry. Approximately, 20 ml slurry were sampled with a plastic syringe and a Viton tubing equipped with a plastic net, placed in a 12-ml Exetainer, and immediately poisoned with 100 μl ZnCl_2_ (7 mol l^−1^) for later analysis of ^29^N_2_ and ^30^N_2_. An additional poisoned slurry sample (~10 ml) was taken, treated with KCl (2 mol l^−1^), centrifuged, filtered and frozen at −20 °C for later analysis of the ^15^NH_4_^+^ fraction in the ammonium pool.

### Anoxic slurry incubation for anammox activity

The presence/absence of sedimentary anammox activity was tested by means of anoxic slurry incubations performed in 12 ml Exetainers. Briefly, the topmost 1.5 cm sediment was collected from a multicorer sediment liner (i.d. 9 cm, height 60 cm) and homogenized in a glass beaker. Then, 2 ml of this sediment was transferred to each of 18 Exetainers, which were filled up with anoxic bottom water. Each vial also received a 4-mm glass bead and was immediately sealed. The slurries were kept homogeneous on a rotating stirrer and preincubated for 12 h to consume all residual O_2_ and NO_3_^−^.

Following preincubation, a degassed 10 mmol l^−1^ stock solution of ^14^NO_3_^−^ and ^15^NH_4_^+^ (99.1 atom %) was injected through the vial septa to reach a final concentration of 150 μmol l^−1^. Nine vials received the ^15^N solution, while nine were left unamended to act as a control. Biological activity was stopped in triplicate samples directly after the addition of substrates by injecting 100 μl ZnCl_2_ (7 mol l^−1^) to each Exetainer. In the same way, triplicate samples were taken after 6 and 12 h incubation at *in situ* temperature (9.5 °C) on the rotating stirrer. The poisoned slurry samples were subsequently analysed for concentrations of ^29^N_2_.

### Infauna quantification and porosity determination

After incubation, the surface sediment layer (~2 cm) of each core was sliced off and sieved sequentially through 1,000 and 40 μm sieves to retrieve samples of macro- and meiofauna. The remaining sediment in each sediment core was sieved through a 1-mm sieve to retrieve deeper burrowing macrofauna individuals. The 40-μm fraction of the sediment from each core was preserved in 4% buffered formalin before extracting the meiofauna, using the method described above. Meiofauna was sorted and counted under a × 50 binocular stereomicroscope. Meiofaunal biomass was calculated according to Olafsson *et al.*^68^ Macrofaunal biomass was determined from the animal dry weight and the carbon content was assumed to be 40% (ref. 69).

One core per treatment was sacrificed for porosity determination. The sediment cores were sliced at 1-cm intervals and porosity was determined from water content and assuming a dry sediment density of 2.6 g cm^−3^. Water content was measured as water loss after drying at 105 °C until constant weight. To use dynamic porosity values for interpretations of O_2_ concentration profiles, the porosity profiles were fitted with polynomial functions^26^.

### Nematode incubations

Exetainer vials were incubated with anoxic water amended with ^15^NO_3_^−^ and increasing numbers of nematodes to test if the animals and/or their symbionts were actively denitrifying. Briefly, 400 ml *in situ* waters were filtered and amended with 1 ml of a Na^15^NO_3_ solution (20 mmol l^−1^ ^15^NO_3_^−^) to reach a concentration of ~50 μmol l^−1^ ^15^NO_3_^−^. The water was degassed for 20 min with Helium 5.0 in a glass bottle equipped with a gas-tight inlet and outlet. One ml of this solution was transferred to each Exetainer (5.9 ml volume) and increasing numbers (5 to 35, in intervals of 5 so that *n*=7) of nematodes were placed in each vial; three additional Exetainers were used as controls with only filtered water and no animals. The nematodes were collected from depths between 1 and 3 cm and extracted alive with the method described above. The Exetainers received additional 3.5 ml of degassed and ^15^NO_3_^−^-amended water and were capped right away. The vials were additionally degassed with Helium for 5 min so that the O_2_ concentration at the beginning of the incubation (tested with a precalibrated microelectrode) was always <1 μmol l^−1^. After 8 h at 9.5 °C, the incubation was terminated by adding 100 μl ZnCl_2_ (7 mol l^−1^) to each of the vials, which were subsequently analysed for concentrations of ^29^N_2_ and ^30^N_2_. Stereomicroscope observations showed that the nematodes were killed immediately after the concentrated ZnCl_2_ solution was added.

### Analyses and calculation

Nitrate and ammonium were determined on a segmented flow autoanalyzer (ALPKEM, Flow Solution IV). Precision was ±0.036 μmol l^−1^ for NH_4_^+^ and ±0.021 μmol l^−1^ for NO_3_^−^. Methane was analysed by headspace analysis on a gas chromatograph equipped with a FID (SRI 8610C). Precision was ±1 nmol l^−1^. Net fluxes of O_2_ and CH_4_ across the sediment–water interface were calculated from the difference in concentration in the water column at the beginning and end of the incubation period.

The concentrations of ^29^N_2_ and ^30^N_2_ in the Exetainers were determined by means of headspace analysis on a gas chromatograph-isotope ratio mass spectrometer^9^. Excess ^29^N_2_ and ^30^N_2_ were used to calculate the N_2_ production over time^31^, and the denitrification rate (*D*_14_) was calculated from the relation:


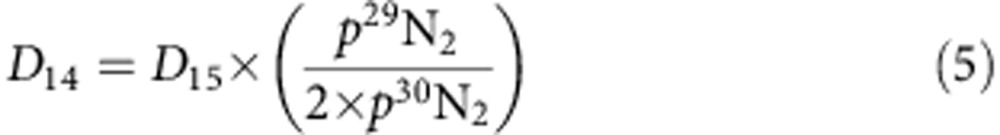


where *p*^29^N_2_ and *p*^30^N_2_ are the production rates of ^29^N_2_ and ^30^N_2_, respectively, and *D*_15_ is calculated as follows:


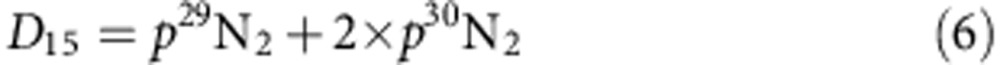


Total denitrification rate was split between denitrification fuelled by water column NO_3_^−^ (*D*_w_) and denitrification coupled to nitrification (*D*_n_) according to equations (7) and (8):


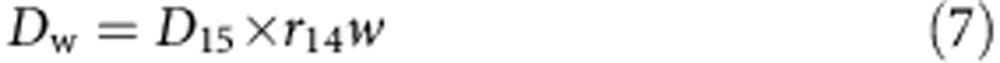


where *r*_14_*w* is the ratio between the concentrations of ^14^NO_3_^−^ and ^15^NO_3_^−^ in the water column;


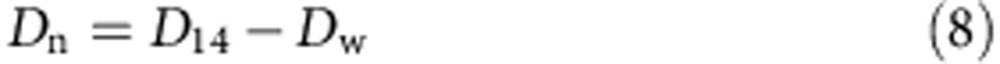


The isotopic composition of NH_4_^+^ was analysed after the conversion of NH_4_^+^ to N_2_ with hypobromite^9^. Analytical precision was 0.5 nmol l^−1^. DNRA rate was calculated as follows:


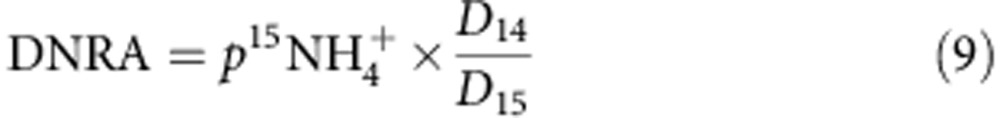


where *p*^15^NH_4_^+^ is the production rate of ^15^N-labelled ammonium during incubation.

### Statistical analyses

Differences between flux/rate calculated for each treatment (LM, HM, LMM, HMM) were tested using parametric (ANOVA) and non-parametric (Kruskal–Wallis) one-way analysis of variance. The CTRL treatment was not compared with the manipulated treatments. Pairwise *post hoc* comparisons among treatments were carried out with the Tukey test. Statistical analyses were performed with Statistica 9.0 (StatSoft). Average values (μmol m^−2^ h^−1^) are reported with associated s.e.m., unless otherwise noted.

## Author contributions

S.B. and F.J.A.N. performed the sampling; S.B., F.J.A.N. and I.K. carried out the experiments; S.B. and M.B. carried out the isotope and gas analysis; F.J.A.N. performed the meiofauna identification. The research was designed by S.B., F.J.A.N., M.B. and V.B. All authors contributed to interpreting the data and writing the paper.

## Additional information

**How to cite this article:** Bonaglia S. *et al.* Meiofauna increases bacterial denitrification in marine sediments. *Nat. Commun.* 5:5133 doi: 10.1038/ncomms6133 (2014).

## Figures and Tables

**Table 1:** Abundances and biomass of meiofauna.

**Treatment**	**Nematoda**	**Ostracoda**			**Harpacticoida**		**Kinorhyncha**	**Oligochaeta**	**Total**
		***Candona neglecta***	***Paracyprideis fennica***	***Heterocyprideis sorbyana***	***Microarthridion littorale***	***Pseudobradya*** **sp.**			
LM	86±19	2±1	8±3	5±2	0±0	0±0	3±2	2±1	106±22
	*1.4±0.3*	*4.1±1.9*	*21.8±7.2*	*13.5±4.3*	*0±0*	*0±0*	*0.8±0.6*	*22.9±8.7*	*64.5±13.6*
HM	718±113	10±4	20±8	15±5	1±2	3±2	9±3	8±3	784±133
	*55.4±8.7*	*16.3±7.3*	*56.0±21.2*	*38.5±12.8*	*1.7±2.5*	*1.0±0.7*	*2.8±0.9*	*84.0±31.7*	*255.6±72.8*
LMM	74±11	2±1	9±1	5±1	0±0	0±0	5±2	1±1	95±11
	*1.2±0.2*	*2.6±2.2*	*23.8±3.6*	*13.7±2.5*	*0±0*	*0±0*	*1.4±0.7*	*10.4±8.5*	*53.0±11.1*
HMM	680±66	9±3	17±8	12±1	1±1	5±2	12±5	9±4	744±81
	*52.4±5.1*	*15.7±4.5*	*47.6±21.8*	*31.2±3.7*	*1.4±1.6*	*1.4±0.7*	*3.5±1.5*	*91.0±42.8*	*244.3±54.1*
Control	462±56	7±1	17±2	12±1	3±1	6±1	10±1	8±1	522±58
(CTRL)	*35.6±4.3*	*11.1±1.2*	*46.2±5.9*	*31.2±3.7*	*3.5±1.0*	*1.7±0.2*	*3.0±0.4*	*78.0±7.4*	*210.2±6.1*

CTRL, unmanipulated sediment cores; HM, high meiofauna; HMM, high meiofauna+macrofauna; LM, low meiofauna; LMM, low meiofauna+macrofauna.

Meiofaunal densities (ind. 10^−3^ m^−2^) are in top rows in roman style and biomasses (μg C 10^−3^ m^−2^) are in second rows in italic style. Values represent average ± s.d. (*n*=5 per treatment).

**Table 2:** Oxygen penetration, oxygen fluxes and diffusivity among treatments.

**Treatment**	**OPD** **(mm)**	***J***_**tot**_ **(μmol m**^−**2**^** h**^−**1**^**)**	***J***_**diff**_ **(μmol m**^−**2**^ **h**^−**1**^**)**	***D***_**S**_ **(cm**^**2**^** s**^−**1**^**)**	***D***_**B**_ **(cm**^**2**^** s**^−**1**^**)**
LM	3.3±0.3	−1,098±34	−882±58	9.9 × 10^−6^	2.4 × 10^−6^
HM	3.7±0.2	−1,321±69	−695±51	10.1 × 10^−6^	9.0 × 10^−6^
LMM	4.2±0.1	−1,211±50	−510±32	10.5 × 10^−6^	14.4 × 10^−6^
HMM	4.7±0.2	−1,412±64	−399±35	10.7 × 10^−6^	27.2 × 10^−6^
Control (CTRL)	5.1±0.1	−1,240±113	−540±68	10.3 × 10^−6^	13.3 × 10^−6^

CTRL, unmanipulated sediment cores; HM, high meiofauna; HMM, high meiofauna+macrofauna; LM; low meiofauna; LMM, low meiofauna+macrofauna; OPD, O_2_ penetration depth.

Average OPD, total benthic O_2_ flux (*J*_tot_) and molecular diffusive O_2_ flux (*J*_diff_)±s.d. (*n*=8 per treatment). *D*_S_ represents molecular diffusivity and *D*_B_ represents biodiffusivity in the top sediment layer. *D*_B_ was calculated from the previous three parameters (see Methods).

**Table 3:** Summary of statistical test results.

**Parameter**	**Analysis**	***P*** **value**	**Differences among treatments**			
			**LM**	**HM**	**LMM**	**HMM**
Meiofauna abundances	*H*_3,19_=14.450	0.002	a	b	a	b
O_2_ penetration depth	*F*_3,23_=10.362	<0.001	a	ab	bc	c
Molecular diffusive O_2_ flux (*J*_diff_)	*F*_3,23_=22.814	<0.001	a	b	c	c
Total O_2_ flux (*J*_tot_)	*F*_3,31_=5.901	0.003	a	b	ab	b
Total denitrification rate (*D*_tot_)	*F*_3,27_=11.371	<0.001	a	b	a	a
Denitrification from water NO_3_^−^ (*D*_w_)	*F*_3,27_=5.707	0.004	a	a	b	b
Coupled nitrification–denitrification (*D*_n_)	*F*_3,27_=11.425	<0.001	a	b	a	a
DNRA rate	*H*_3,27_=13.381	0.004	a	a	b	b
CH_4_ flux	*H*_3,31_=24.662	<0.001	a	a	b	b

DNRA, dissimilatory nitrate reduction to ammonium; HM, high meiofauna; HMM, high meiofauna+macrofauna; LM, low meiofauna; LMM, low meiofauna+macrofauna.

One-way parametric (*F* values) and non-parametric Kruskal–Wallis analysis of variance (*H* values) among the different treatments. Pairwise comparison was performed by means of Tukey test. Different letters represent significant differences (*P*<0.05), while the same letter represents no significant differences (*P*>0.05) among treatments.

## References

[b1] Cardinale B. J. Biodiversity improves water quality through niche partitioning. Nature 472, 86–89 (2011). 21475199 10.1038/nature09904

[b2] Dodds W. K. *et al.* Eutrophication of US freshwaters: analysis of potential economic damages. Environ. Sci. Technol. 43, 12–19 (2009). 19209578 10.1021/es801217q

[b3] Galloway J. N. *et al.* Nitrogen cycles: past, present, and future. Biogeochemistry 70, 153–226 (2004).

[b4] Seitzinger S. *et al.* Denitrification across landscapes and waterscapes: a synthesis. Ecol. Appl. 16, 2064–2090 (2006). 17205890 10.1890/1051-0761(2006)016[2064:dalawa]2.0.co;2

[b5] Burgin A. J. & Hamilton S. K. Have we overemphasized the role of denitrification in aquatic ecosystems? A review of nitrate removal pathways. Front. Ecol. Environ. 5, 89–96 (2007).

[b6] Thamdrup B. New pathways and processes in the global nitrogen cycle. Annu. Rev. Ecol. Evol. Syst. 43, 407–428 (2012).

[b7] Dong L. F. *et al.* Dissimilatory reduction of nitrate to ammonium, not denitrification or anammox, dominates benthic nitrate reduction in tropical estuaries. Limnol. Oceanogr. 56, 279–291 (2011).

[b8] Gardner W. S. *et al.* Nitrogen fixation and dissimilatory nitrate reduction to ammonium (DNRA) support nitrogen dynamics in Texas estuaries. Limnol. Oceanogr. 51, 558–568 (2006).

[b9] Bonaglia S., Deutsch B., Bartoli M., Marchant H. K. & Brüchert V. Seasonal oxygen, nitrogen and phosphorus benthic cycling along an impacted Baltic Sea estuary: regulation and spatial patterns. Biogeochemistry 119, 139–160 (2014).

[b10] Elmgren R. Eutrophication: political backing to save the Baltic Sea. Nature 487, 432–432 (2012). 22836990 10.1038/487432d

[b11] Loreau M. Linking biodiversity and ecosystems: towards a unifying ecological theory. Phil. Trans. R. Soc. B 365, 49–60 (2010). 20008385 10.1098/rstb.2009.0155 PMC2842700

[b12] Kristensen E. & Kostka J. E. inInteractions Between Macro- and Microorganisms in Marine Sediments (eds Kristensen E., Haese R. R., Kostka J. E. 125–158AGU (2005).

[b13] Aller R. C. Bioturbation and remineralization of sedimentary organic matter: effects of redox oscillation. Chem. Geol. 114, 331–345 (1994).

[b14] Karlson K., Bonsdorff E. & Rosenberg R. The impact of benthic macrofauna for nutrient fluxes from Baltic Sea sediments. Ambio 36, 161–167 (2007). 17520929 10.1579/0044-7447(2007)36[161:tiobmf]2.0.co;2

[b15] Stief P. Stimulation of microbial nitrogen cycling in aquatic ecosystems by benthic macrofauna: mechanisms and environmental implications. Biogeosciences 10, 7829–7846 (2013).

[b16] Bonaglia S. *et al.* Effect of reoxygenation and *Marenzelleria* spp. bioturbation on Baltic Sea sediment metabolism. Mar. Ecol. Prog. Ser. 482, 43–55 (2013).

[b17] Nizzoli D. *et al.* Implications for oxygen, nutrient fluxes and denitrification rates during the early stage of sediment colonisation by the polychaete *Nereis* spp. in four estuaries. Estuar. Coast. Shelf Sci. 75, 125–134 (2007).

[b18] Pelegri S. P. & Blackburn T. H. Effect of bioturbation by *Nereis* sp., *Mya arenaria* and *Cerastoderma* sp. on nitrification and denitrification in estuarine sediments. Ophelia 42, 289–299 (1995).

[b19] Nascimento F. J. A., Näslund J. & Elmgren R. Meiofauna enhances organic matter mineralization in soft sediment ecosystems. Limnol. Oceanogr. 57, 338–346 (2012).

[b20] Näslund J., Nascimento F. J. A. & Gunnarsson J. S. Meiofauna reduces bacterial mineralization of naphthalene in marine sediment. ISME J. 4, 1421–1430 (2010). 20463764 10.1038/ismej.2010.63

[b21] Coull B. C. Role of meiofauna in estuarine soft-bottom habitats. Aust. J. Ecol. 24, 327–343 (1999).

[b22] Danovaro R. *et al.* The first metazoa living in permanently anoxic conditions. BMC Biol. 8, 30 (2010). 20370908 10.1186/1741-7007-8-30 PMC2907586

[b23] Glud R. N., Gundersen J. K., Jørgensen B. B., Revsbech N. P. & Schulz H. D. Diffusive and total oxygen uptake of deep-sea sediments in the eastern South Atlantic Ocean: in situ and laboratory measurements. Deep Sea Res. Pt. I 41, 1767–1788 (1994).

[b24] Rysgaard S., Christensen P. B., Sorensen M. V., Funch P. & Berg P. Marine meiofauna, carbon and nitrogen mineralization in sandy and soft sediments of Disko Bay, West Greenland. Aquat. Microb. Ecol. 21, 59–71 (2000).

[b25] Levin L. A. Oxygen minimum zone benthos: adaptation and community response to hypoxia. Oceanogr. Mar. Biol. Annu. Rev. 41, 1–45 (2003).

[b26] Berg P., Rysgaard S., Funch P. & Sejr M. K. Effects of bioturbation on solutes and solids in marine sediments. Aquat. Microb. Ecol. 26, 81–94 (2001).

[b27] Aller R. C. & Aller J. Y. Meiofauna and solute transport in marine muds. Limnol. Oceanogr. 37, 1018–1033 (1992).

[b28] Høgslund S., Revsbech N. P., Cedhagen T., Nielsen L. P. & Gallardo V. A. Denitrification, nitrate turnover, and aerobic respiration by benthic foraminiferans in the oxygen minimum zone off Chile. J. Exp. Mar. Biol. Ecol. 359, 85–91 (2008).

[b29] Risgaard-Petersen N. *et al.* Evidence for complete denitrification in a benthic foraminifer. Nature 443, 93–96 (2006). 16957731 10.1038/nature05070

[b30] Ankar S. & Elmgren R. The benthic macro- and meiofauna of the Askö-Landsort Area (northern Baltic proper): a stratified random sampling survey. Contributions from the Askö Laboratory, University of Stockholm11, 1–115 (1976).

[b31] Nielsen L. P. Denitrification in sediment determined from nitrogen isotope pairing. FEMS Microbiol. Ecol. 86, 357–362 (1992).

[b32] Hermelin J. O. R. Distribution of Holocene benthic foraminifera in the Baltic Sea. J. Foramin. Res. 17, 62–73 (1987).

[b33] Hentschel U., Berger E. C., Bright M., Felbeck H. & Ott J. A. Metabolism of nitrogen and sulfur in ectosymbiotic bacteria of marine nematodes (Nematoda, Stilbonematinae). Mar. Ecol. Prog. Ser. 183, 149–158 (1999).

[b34] Finlay B. J., Span A. S. W. & Harman J. M. P. Nitrate respiration in primitive eukaryotes. Nature 303, 333–336 (1983).

[b35] Zumft W. G. Cell biology and molecular basis of denitrification. Microbiol. Mol. Biol. Rev. 61, 533–616 (1997). 9409151 10.1128/mmbr.61.4.533-616.1997 PMC232623

[b36] Canfield D. E., Kristensen E. & Thamdrup B. inAquatic Geomicrobiology (eds Canfield D. E., Kristensen E., Thamdrup B. )205–267Academic Press (2005).

[b37] Jenkins M. C. & Kemp W. M. The coupling of nitrification and denitrification in two estuarine sediments. Limnol. Oceanogr. 29, 609–619 (1984).

[b38] Prast M., Bischoff A. A., Waller U., Amann R. & Berninger U. G. Effect of ciliates on nitrification and nitrifying bacteria in Baltic Sea sediments. Mar. Ecol. Prog. Ser. 350, 55–61 (2007).

[b39] Montagna P. A. *In situ* measurement of meiobenthic grazing rates on sediment bacteria and edaphic diatoms. Mar. Ecol. Prog. Ser. 18, 119–130 (1984).

[b40] Alongi D. M. Microbes, meiofauna, and bacterial productivity on tubes constructed by the polychaete *Capitella capitata*. Mar. Ecol. Prog. Ser. 23, 207–208 (1985).

[b41] Danovaro R. *et al.* Exponential decline of deep-sea ecosystem functioning linked to benthic biodiversity loss. Curr. Biol. 18, 1–8 (2008). 18164201 10.1016/j.cub.2007.11.056

[b42] Ferris H., Venette R. C., van der Meulen H. R. & Lau S. S. Nitrogen mineralization by bacterial-feeding nematodes: verification and measurement. Plant Soil 203, 159–171 (1998).

[b43] Moens T. *et al.* Do nematode mucus secretions affect bacterial growth? Aquat. Microb. Ecol. 40, 77–83 (2005).

[b44] Moens T., Bergtold M. & Traunspurger W. inFreshwater Nematodes: Ecology and Taxonomy (eds Eyulaem A., Andrassy I., Traunspurger W. 105–131CAB International Publishing (2006).

[b45] Bott T. L. & Borchardt M. A. Grazing of protozoa, bacteria, and diatoms by meiofauna in lotic epibenthic communities. J. N. Am. Benthol. Soc. 18, 499–513 (1999).

[b46] Branch G. M. & Pringle A. The impact of the sand prawn *Callianassa kraussi* Stebbing on sediment turnover and on bacteria, meiofauna, and benthic microflora. J. Exp. Mar. Biol. Ecol. 107, 219–235 (1987).

[b47] Olafsson E. Do macrofauna structure meiofauna assemblages in marine soft-bottoms? A review of experimental studies. Vie Milieu 53, 249–265 (2003).

[b48] Nascimento F. A., Karlson A. L., Näslund J. & Elmgren R. Diversity of larger consumers enhances interference competition effects on smaller competitors. Oecologia 166, 337–347 (2011). 21161548 10.1007/s00442-010-1865-0 PMC3094539

[b49] Aller R. C. & Yingst J. Y. Effects of the marine deposit-feeders *Heteromastus filiformis* (Polychaeta), *Macoma balthica* (Bivalvia), and *Tellina texana* (Bivalvia) on averaged sedimentary solute transport, reaction rates, and microbial distributions. J. Mar. Res. 43, 615–645 (1985).

[b50] Joye S. B. & Hollibaugh J. T. Influence of sulfide inhibition of nitrification on nitrogen regeneration in sediments. Science 270, 623–625 (1995).

[b51] Jørgensen B. B. & Nelson D. C. Sulfide oxidation in marine sediments: geochemistry meets microbiology. Geol. S. Am. S. 379, 63–81 (2004).

[b52] Ferguson A. J. P. & Eyre B. D. Seasonal discrepancies in denitrification measured by isotope pairing and N_2_:Ar techniques. Mar. Ecol. Prog. Ser. 350, 19–27 (2007).

[b53] Giere O. Meiobenthology: The Microscopic Motile Fauna of Aquatic Sediments 2nd edn Springer-Verlag (2009).

[b54] Michaud E., Desrosiers G., Mermillod-Blondin F., Sundby B. & Stora G. The functional group approach to bioturbation: II. The effects of the *Macoma balthica* community on fluxes of nutrients and dissolved organic carbon across the sediment–water interface. J. Exp. Mar. Biol. Ecol. 337, 178–189 (2006).

[b55] Risgaard-Petersen N., Nielsen L. P., Rysgaard S., Dalsgaard T. & Meyer R. L. Application of the isotope pairing technique in sediments where anammox and denitrification coexist. Limnol. Oceanogr. Methods 1, 63–73 (2003).

[b56] Stief P. & Eller G. The gut microenvironment of sediment-dwelling *Chironomus plumosus* larvae as characterised with O_2_, pH, and redox microsensors. J. Comp. Physiol. B 176, 673–683 (2006). 16721623 10.1007/s00360-006-0090-y

[b57] Harris J. The presence, nature, and role of gut microflora in aquatic invertebrates: a synthesis. Microb. Ecol. 25, 195–231 (1993). 24189919 10.1007/BF00171889

[b58] Brauman A., Kane M. D., Labat M. & Breznak J. A. Genesis of acetate and methane by gut bacteria of nutritionally diverse termites. Science 257, 1384–1387 (1992). 17738281 10.1126/science.257.5075.1384

[b59] Ngugi D. K. & Brune A. Nitrate reduction, nitrous oxide formation, and anaerobic ammonia oxidation to nitrite in the gut of soil-feeding termites (*Cubitermes* and *Ophiotermes* spp.). Environ. Microbiol. 14, 860–871 (2012). 22118414 10.1111/j.1462-2920.2011.02648.x

[b60] Childress J. J. *et al.* A methanotrophic marine molluscan (Bivalvia, Mytilidae) symbiosis: Mussels fueled by gas. Science 233, 1306–1308 (1986). 17843358 10.1126/science.233.4770.1306

[b61] Glud R. N. *et al.* Nitrogen cycling in a deep ocean margin sediment (Sagami Bay, Japan). Limnol. Oceanogr. 54, 723–734 (2009).

[b62] Glover A. G. & Smith C. R. The deep-sea floor ecosystem: current status and prospects of anthropogenic change by the year 2025. Environ. Conserv. 30, 219–241 (2003).

[b63] Guerrini A., Colangelo M. A. & Ceccherelli V. U. Recolonisation patterns of meiobenthic communities in brackish vegetated and unvegetated habitats after induced hypoxia/anoxia. Hydrobiologia 375-376, 73–87 (1998).

[b64] Blomqvist S. & Larsson U. Detrital bedrock elements as tracers of settling resuspended particulate matter in a coastal area of the Baltic Sea. Limnol. Oceanogr. 39, 880–896 (1994).

[b65] Berg P., Risgaard-Petersen N. & Rysgaard S. Interpretation of measured concentration profiles in sediment pore water. Limnol. Oceanogr. 43, 1500–1510 (1998).

[b66] Iversen N. & Jørgensen B. B. Diffusion coefficients of sulfate and methane in marine sediments: Influence of porosity. Geochim. Cosmochim. Acta 57, 571–578 (1993).

[b67] Schulz H. D. inMarine Geochemistry (eds Schulz H. D., Zabel M. 73–124Springer (2006).

[b68] Olafsson E., Modig H. & Van de Bund W. J. Species specific uptake of radio-labelled phytodetritus by benthic meiofauna from the Baltic Sea. Mar. Ecol. Prog. Ser. 177, 63–72 (1999).

[b69] Karlson A. M. L., Nascimento F. J. A., Näslund J. & Elmgren R. Higher diversity of deposit-feeding macrofauna enhances phytodetritus processing. Ecology 91, 1414–1423 (2010). 20503873 10.1890/09-0660.1

